# Benthic Algal Community Structures and Their Response to Geographic Distance and Environmental Variables in the Qinghai-Tibetan Lakes With Different Salinity

**DOI:** 10.3389/fmicb.2018.00578

**Published:** 2018-03-27

**Authors:** Jian Yang, Hongchen Jiang, Wen Liu, Beichen Wang

**Affiliations:** State Key Laboratory of Biogeology and Environmental Geology, China University of Geosciences, Wuhan, China

**Keywords:** benthic algal community, salinity, geographic distance, lakes, Qinghai-Tibetan Plateau

## Abstract

Uncovering the limiting factors for benthic algal distributions in lakes is of great importance to understanding of their role in global carbon cycling. However, limited is known about the benthic algal community distribution and how they are influenced by geographic distance and environmental variables in alpine lakes. Here, we investigated the benthic algal community compositions in the surface sediments of six lakes on the Qinghai-Tibetan Plateau (QTP), China (salinity ranging from 0.8 to 365.6 g/L; pairwise geographic distance among the studied lakes ranging 8–514 km) employing an integrated approach including Illumina-Miseq sequencing and environmental geochemistry. The results showed that the algal communities of the studied samples were mainly composed of orders of *Bacillariales*, *Ceramiales*, *Naviculales*, *Oscillatoriales*, *Spirulinales*, *Synechococcales*, and *Vaucheriales*. The benthic algal community compositions in these QTP lakes were significantly (*p* < 0.05) correlated with many environmental (e.g., dissolved inorganic and organic carbon, illumination intensity, total nitrogen and phosphorus, turbidity and water temperature) and spatial factors, and salinity did not show significant influence on the benthic algal community structures in the studied lakes. Furthermore, geographic distance showed strong, significant correlation (*r* = 0.578, *p* < 0.001) with the benthic algal community compositions among the studied lakes, suggesting that spatial factors may play important roles in influencing the benthic algal distribution. These results expand our current knowledge on the influencing factors for the distributions of benthic alga in alpine lakes.

## Introduction

The littoral zone of lakes is one of the most productive ecosystems on Earth, and it plays significant roles in the functioning (e.g., carbon cycling) of lacustrine ecosystems (Howard-Williams and Lenton, 1975; Strayer and Likens, 1986; Stoffels et al., 2005). Such inshore habitat hosts diverse algal communities, which are important contributors for primary production in aquatic ecosystems (Nozaki, 2001; Nozaki et al., 2003; Mooij et al., 2005; Nõges et al., 2010). Generally, planktonic algal community has dominant contribution for primary productions in lakes (Bryant and Frigaard, 2006; Reinfelder, 2011; Althouse et al., 2014). Recent studies reported that littoral benthic algae also make significant or dominant contribution to total primary production within certain lakes (Vadeboncoeur et al., 2001; Ask et al., 2009; Althouse et al., 2014). The primary production is commonly mediated by algal community composition, because different algal species have distinct carbon fixation capability (Reinfelder, 2011). Therefore, studies on the distribution and composition of benthic alga in lakes are of great importance to understanding of carbon cycling in lacustrine ecosystems.

Previous studies reported that planktonic algal distribution was often affected by many environmental factors such as salinity (Huang et al., 2014; Liu X. et al., 2016), pH (Kenneth, 2002), nutrient (Stelzer and Lamberti, 2001; Wyatt et al., 2010), and light (Lange et al., 2011). Furthermore, geographic distance influence on the algal (e.g., diatom) distribution was also reported in some freshwater aquatic ecosystems (e.g., river, wetlands, streams) (Astorga et al., 2012; Wetzel et al., 2012; Goldenberg Vilar et al., 2014), However, studies are limited on benthic algal community composition and distribution in saline lakes. So it is poorly known whether geographic distance and environmental factors can influence the benthic algal community compositions among saline and hypersaline lakes.

The Qinghai-Tibetan Tibetan (QTP) host thousands of lakes (more than 1000 lakes with surface area >1 km^2^) with salinity ranging from 0.1 to 426.3 g/L (Zheng, 1997). Many previous studies showed that salinity was the most important factor influencing microbial distribution and function in the QTP lakes (Wu et al., 2006; Jiang et al., 2007, 2012; Xing et al., 2009; Wang et al., 2011; Liu et al., 2013; Huang et al., 2014; Liu Y. et al., 2016; Yang et al., 2016b). However, little is known about the distribution of benthic alga in the QTP lakes and how they respond to the changes of environmental variables (e.g., salinity) and geographic distance among lakes. In this study, the major objectives were to examine the benthic algal community compositions in the QTP lakes and evaluate how they were influenced by geographic distance and environmental factors. In order to fulfill above objectives, a total of 18 littoral sediments were collected from six lakes (triplicates were applied for each of the studied lakes) in the QTP. The pairwise distances of the sampled lakes were 8–514 km. Illumina-Miseq sequencing was employed to investigate the plastid 23S rRNA genes of the benthic algal community compositions in these lake sediments.

## Materials and Methods

### Sample Collection

Sampling cruise was carried out in May 2016. Six Qinghai-Tibetan lakes (Supplementary Figure S1) were selected for this study: Erhai Lake (EHL) is a freshwater lake; Qinghai Lake (QHL) and Tuosu Lake (TSL) are saline lakes; Gahai Lake (GHL), Xiaochaidan Lake (XCDL) and Chaka Lake (CKL) are hypersaline lakes (Yang et al., 2013). The TSL, GHL, and XCDL are located in the Qaidam Basin (QB) hinterland, while the CKL is on the QB fringe and the other lakes (i.e., EHL, QHL) are situated out of the QB (Zheng, 1997) (Supplementary Figure S1). In this study, a total of 18 sampling sites (triplicates from each of the studied lakes) were sampled. At each sampling site, the pH and temperature of lake surface water were measured with a portable SX711 pH meter (SANXIN, Shanghai, China); water turbidity was analyzed using a turbidity meter (HANNA, Romania); In-situ illumination intensity was determined by using a TES1335 light meter (TES, Taiwan, China). Water samples (∼20 mL) for measurements of major ions were collected after filtrating through 0.2-μm Nuclepore filters (Whatman, United Kingdom); water samples (∼20 mL) for measurements of dissolved organic carbon (DOC) were filtered through combusted 0.7-μm Whatman GF/F filters and the resulting filtrate was collected into a dark glass vial pre-acidified with concentrated phosphoric acid (∼40 μL); water samples (∼40 mL each) for measurements of dissolved inorganic carbon (DIC), water samples for total nitrogen (TN) and total phosphorus (TP) were collected into 40 mL dark glass vials without air bubbles, supplemented with saturated mercury chloride (∼40 μL) before covering lid. Additionally, 500 mL water samples were filtered through 0.7-μm glass fiber filters (Whatman, United Kingdom), and the filters were stored in dry ice collected for the measurements of chlorophyll a (Chl-a) concentration. Surface sediments (∼0–1 cm) were collected using a grab-bucket collection sampler in the littoral zones of lakes with water depth of ∼1 meter. The surface sediments were then collected into 50 mL sterilized tubes using sterile spoons for DNA samples. The DNA and Chl-a samples were stored in dry ice in the field and during transportation and then were transferred to a -80°C freezer in the laboratory until further analyses. Other samples (e.g., water samples for major ions, DOC, DIC, TN, and TP) were stored at 4°C during transportation and were analyzed immediately after arrival in laboratory.

### Laboratory Geochemical Analyses

Cation and anion concentrations (e.g., K^+^, Na^+^, Ca^2+^, Mg^2+^, SO_4_^2-^, Cl^-^) of the lake waters were measured by using ion chromatography (Dionex DX-600, United States). Salinity was calculated by summing up the concentrations of six major ions including K^+^, Na^+^, Ca^2+^, Mg^2+^, SO_4_^2-^, and Cl^-^. DOC and TN concentrations were measured on a multi N/C 2100S analyzer (Analytik Jena, Germany). DIC was measured by mean of the potentiometric acid titration method (Bradshaw et al., 1981). TP was analyzed by phosphormolybdic acid colorimetry (Neal et al., 2000). Chl-a was measured using a fluorospectrophotometer (Shimadzu Corp., Japan) following an overnight freeze-thaw extraction in 90% acetone (Liu et al., 2006).

### DNA Extraction and Sequencing

Total DNA was extracted from 0.5 g sediment samples using the Fast DNA SPIN Kit for Soil (MP Biomedical, United States). The extracted DNA was amplified with a universal algal 23S rRNA gene primer set p23SrV_f1 and p23SrV_r1, and the detailed PCR conditions were described in a previous study (Sherwood and Presting, 2007). Briefly, a unique 12-bp barcode sequence was added between the sequencing adapter and reverse primer to differentiate among samples. Triplicate PCR reactions for each sample were conducted and the resulting successful PCR products were purified using a DNA Gel Extraction Kit (Axygen, United States). The PCR amplicons (∼400 bp) from each sample were pooled with equimolar concentrations and then were sequenced by using an Illumina-Miseq platform (paired-ends sequencing of 2 × 250 bp) (Caporaso et al., 2012).

### Raw 23S rRNA Gene Sequences Processing and Statistical Analyses

The raw 23S rRNA gene sequences were processed following the pipeline coupling USEARCH (Edgar, 2013) and QIIME (Caporaso et al., 2010) software. The paired reads were joined with FLASH (fast length adjustment of short reads) using default setting (Magoč and Salzberg, 2011). Forward and reverse primers were removed from the joined reads. The remaining reads were then de-multiplexed and quality filtered using QIIME v1.9.0 with *split_libraries_fastq.py* script (Caporaso et al., 2010). Briefly, reads having more than three consecutive low quality (Phred quality score <30) bases were removed, and reads containing ambiguous base were discarded, as well as reads comprising consecutive high quality bases less than 75% of the total read length were culled out. Chimera checking was performed using the UCHIME module with *de novo* method in USEARCH (Edgar et al., 2011). Singleton and read length less than 200 were discarded, and operational taxonomic units (OTUs ) were defined at the 97% cutoff (Steven et al., 2012) by using the UCLUST algorithm (Edgar, 2010). OTU representative sequences were then selected and their taxonomy were assigned using *parallel_assign_taxonomy_blast.py* with default set (sequences similarity >90% and blasted exception value<10^-3^) against the SILVA 128 LSU database in the QIIME program. Sequences failing to be assigned into *Cyanobacteria* and eukaryotic algae were removed. In order to validate these assignments of taxonomy, OTU representative sequences were locally BLASTed in NCBI database^1^. The BLASTed results were provided in Supplementary Table S1. The final OTU table was rarefied to equal sequence number (*n* = 8843) for each sample with 1000 times, and then alpha diversity was calculated at the 97% identity level in QIIME. A variety of alpha diversity indices were calculated including Simpson, Shannon, Equitability and Chao1.

All environmental variables in this study were normalized to values ranged between 1 and 100 as described previously (Yang et al., 2016a). The non-metric dimensional scaling (NMDS) ordination with 500 random starts were performed to depict the difference of algal community compositions among lakes based on the Bray-Curtis dissimilarity using the package “vegan.” Cluster analysis was performed according to the Bray-Curtis dissimilarity among samples using PAST software^2^. Simple Mantel tests were performed to assess the Spearman’s correlations between algal community compositions and geographic distance/environmental variables by using the “vegan” package. Geographical distances among sampling sites were calculated based on the GPS locations of each sites using Euclidean method in PAST software (Supplementary Table S2). Canonical correspondence analysis (CCA) was also performed to explore the relationships between algal communities and environmental and spatial variables. Before the CCA, a set of spatial variables were generated through the method of principal coordinates of neighbor matrices (PCNM) analysis according to the longitude and latitude coordinates of the sampling sites (Borcard and Legendre, 2002). Subsequently, we used a forward selection procedure to select environmental and spatial variables through the ‘*ordiR2step*’ function in R package “vegan” (Blanchet et al., 2008). Only significant (*p* < 0.05) environmental and spatial variables were shown in the CCA ordination.

In order to discern the difference between benthic and planktonic algal community composition in lakes, planktonic algal 23S rRNA gene sequences were collected from the two published studies (Steven et al., 2012; Liu X. et al., 2016). To avoid any bias resulting from different primers, only 23S rRNA gene sequences derived from the same primer set (p23SrV_f1 and p23SrV_r1) and the same PCR protocol were included in this analysis. Sequences were processed according to the procedures described above. NMDS ordination with 500 random starts were conducted to discern the difference between benthic (this study) and planktonic (previous studies) algal community compositions in lakes according to the Bray-Curtis dissimilarity. In addition, the dominant OTU representative sequences of *Cyanobacteria* (average relative abundance >0.1%) were selected to perform BLAST^3^ against available 23S rRNA genes in the GenBank. Meanwhile, their closest references were retrieved for constructing phylogenic tree. All the OTU representative sequences were aligned with their references by using Clustal W implemented in the Bioedit program. Maximum-likelihood tree was constructed from the representative cyanobacterial 23S rRNA sequences and their references by using the MEGA 6.0.

### Nucleotide Sequence Accession Numbers

The sequence data generated in this study were deposited at the Sequence Read Archive (SRA) in the National Center for Biotechnology Information (NCBI) under the BioProject PRJNA376846 with accession no. SRP101378.

## Results

### Environmental Parameters of the Studied Samples

The studied lakes have a large range of environmental parameters (Supplementary Table S3). For example, the salinity was 0.8–365.6 g/L and the pH was 7.4–9.2; DOC was 1.9–26.3 mM and DIC was 6.2–29.8 mM; turbidity was 0.6–17.4 NTU (Nephelometric Turbidity Unit) and the concentration of Chl-a was 0.1–21.4 μg/L; TN and TP ranged 97.7–441.1 and 3.8–13.7 μM, respectively.

### Benthic Algal Community Composition

**FIGURE 1:**
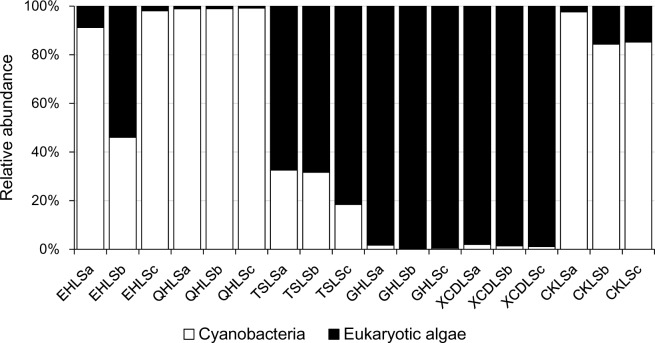
Proportions of Cyanobacteria and eukaryotic algae in the studied lake samples.

**FIGURE 2:**
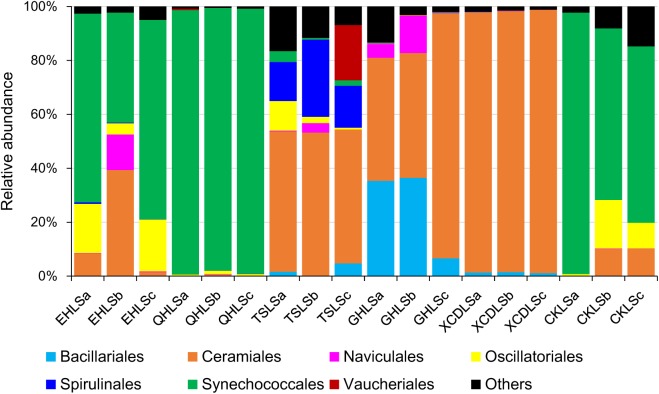
Relative abundance of algal sequences at the order level in the studied lake samples.

A total of 2, 244, 853 qualified sequence reads were obtained after data processing, and 89.1% of total qualified sequence reads (1, 999, 487 sequence reads) were assigned to *Cyanobacteria* and eukaryotic algae. The algal sequences per sample ranged from 8,843 to 764,193 with an average of 101, 082. Alpha diversity indices of the studied samples were summarized in **Table 1**. The number of the observed algal OTUs of the studied samples ranged 9.0–65.2 with Shannon indices and Chao 1 being 0.6–3.9 and 9.0–83.4, respectively (**Table 1**). These alpha-diversity indices were not significantly correlated with any environmental parameters of the studied lakes (data not shown). The relative abundances of *Cyanobacteria* and eukaryotic algae ranged 0.2–99.2 and 0.8–99.8% among the samples, respectively (**Figure 1**). *Cyanobacteria* was dominant (relative abundance >45%) in the samples of EHL, QHL, and CKL, whereas eukaryotic algae largely dominated (relative abundance >60%) in the samples of TSL, GHL and XCDL (**Figure 1**). The algal communities of the studied samples were composed of seven dominant (relative abundance >5% at least in one sample) orders (i.e., *Bacillariales*, *Ceramiales*, *Naviculales*, *Oscillatoriales*, *Spirulinales*, *Synechococcales*, and *Vaucheriales*) (**Figure 2**). Algal sequences belonging to *Synechococcales* were dominant in the samples of EHL, QHL, and CKL, whereas *Ceramiales* sequences dominated in the samples of TSL, GHL, and XCDL (**Figure 2**).

**Table 1:** Alpha diversity of the studied samples (a, b, and c indicate replicate samples).

Sample	Total sequences	Algal sequences	Observed OTUs	Simpson	Shannon	Equitability	Chao1
EHLSa	90131	80292	65.2	0.8	3.3	0.5	81.5
EHLSb	172207	143090	61.8	0.8	2.9	0.5	78.5
EHLSc	169488	145123	63.4	0.9	3.4	0.6	83.4
QHLSa	379250	375963	52.7	0.4	1.2	0.2	74.1
QHLSb	69241	68707	49.0	0.4	1.3	0.2	63.0
QHLSc	774594	764193	40.1	0.3	1.0	0.2	59.8
TSLSa	16018	14269	31.0	0.7	2.5	0.5	32.4
TSLSb	17156	16902	24.3	0.7	2.3	0.5	25.0
TSLSc	9057	8843	22.0	0.7	2.3	0.5	22.0
GHLSa	22469	16270	33.8	0.7	2.0	0.4	37.1
GHLSb	19691	18299	22.0	0.6	1.7	0.4	25.1
GHLSc	19467	11671	19.7	0.2	0.6	0.1	20.2
XCDLSa	12405	11996	9.9	0.5	1.2	0.4	9.9
XCDLSb	10727	10665	9.0	0.5	1.2	0.4	9.0
XCDLSc	25109	24899	10.8	0.5	1.2	0.3	11.6
CKLSa	149077	86339	23.8	0.4	1.1	0.2	30.2
CKLSb	123130	75456	36.0	0.9	3.9	0.8	41.8
CKLSc	165636	126510	32.6	0.9	3.3	0.7	45.1

### Influence of Environmental and Spatial Variables on Algal Distribution

**FIGURE 3:**
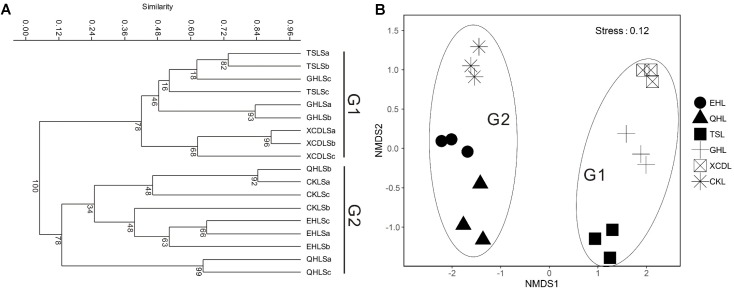
Clustering **(A)** and NMDS ordination **(B)** analysis on the basis of Bray-Curtis dissimilarities among the studied lake samples. G1: the group of samples from lakes in the Qaidam Basin hinterland; G2: the group of samples from the fringe (CKL) and out (EHL and QHL) of the Qaidam Basin.

**FIGURE 4:**
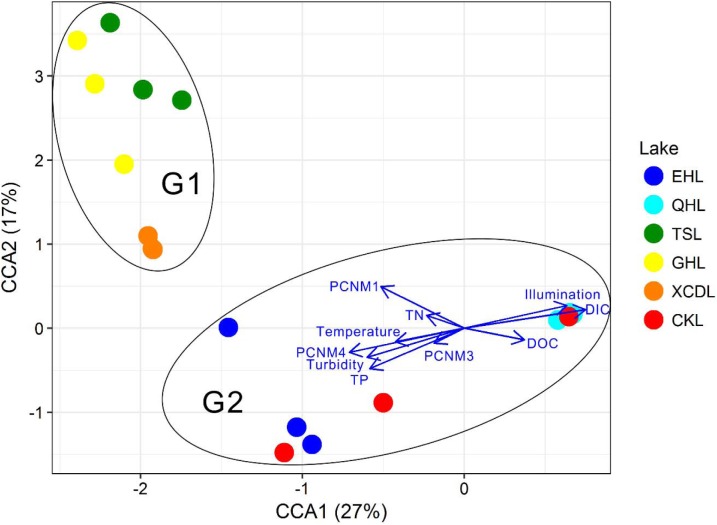
CCA ordination showing the benthic algal community composition in relation to significant environmental and spatial factors (*p* < 0.05).

**FIGURE 5:**
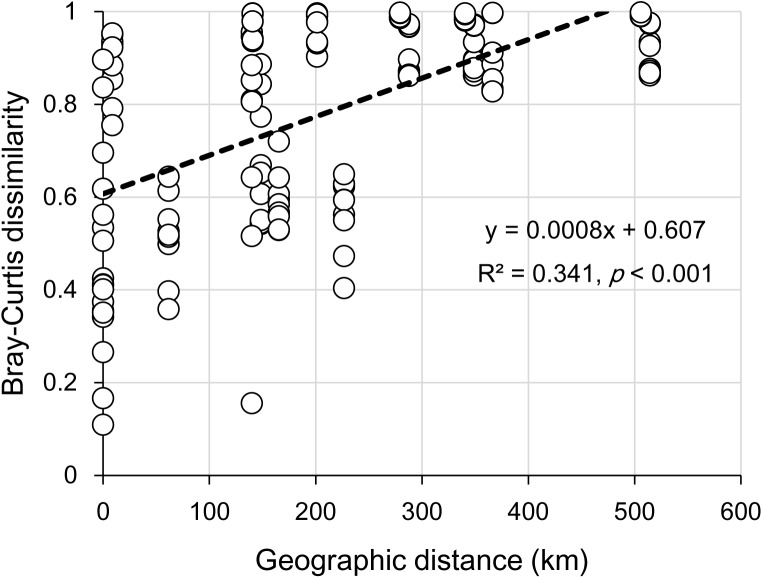
Linear relationship between geographic distance and the Bray-Curtis dissimilarities of the benthic algal communities among the studied lake samples.

The clear geographic patters of benthic algal community were observed among the studied lakes (**Figure 3**): the benthic algal communities in the lakes (i.e., TSL, GHL, XCDL) within the Qaidam Basin (QB) hinterland were grouped into the G1 cluster, and those from EHL, QHL, and CKL were grouped into the G2 cluster (**Figure 3A**). Similar grouping patterns were also observed in the NMDS and CCA ordination, which showed that the distributions of algal communities in TSL, GHL, and XCDL (G1) were separated from those of EHL, QHL and CKL (G2) along the axis NMDS1 (**Figures 3B**, **4**). The CCA result also indicated that many local environmental (i.e., DOC, DIC, illumination intensity, TN, TP, turbidity, water temperature) and spatial (i.e., PCNM1, PCNM3, and PCNM4) variables significantly (*p* < 0.05) affect the benthic algal distribution in the studied lakes (**Figure 4**). Furthermore, Mantel tests showed that the algal community compositions of the studied samples were significantly (*p* < 0.05) correlated with DIC, DOC, geographic distance, pH, salinity and water temperature (**Table 2**). Geographic distance possessed higher correlation coefficient than other environmental variables (e.g., salinity, pH, etc.). Linear analysis gave a R^2^ of 0.341 (*p* < 0.001) between the dissimilarities of benthic algal community among lakes and geographic distances (**Figure 5**).

**Table 2:** Mantel test-based correlations between algal community compositions and the measured environmental factors in the studied samples.

Factors	r	*p*
Chlorophyll-a	0.129	0.094
DIC	**0.350**	**0.004**
DOC	**0.446**	**0.001**
Geographic distance	**0.578**	**<0.001**
Illumination intensity	0.101	0.122
pH	**0.161**	**0.048**
Salinity	**0.214**	**0.033**
TN	0.113	0.095
TP	0.075	0.184
Turbidity	0.153	0.054
Water temperature	**0.178**	**0.043**

*r: Spearman correlation coefficient. Bold face indicate a significant correlation with p < 0.05.*

### Comparisons Between Benthic and Planktonic Algal Community Compositions in Lakes

**FIGURE 6:**
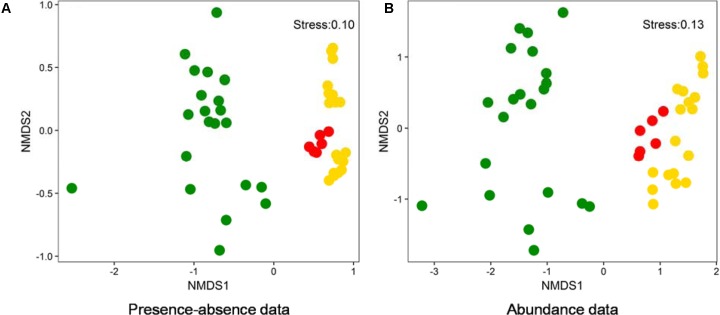
NMDS ordination analysis based on presence-absence **(A)** and abundance data **(B)** showing the algal community compositions of different lake samples collected from this study and two previous studies (Steven et al., 2012; Liu X. et al., 2016). Green solid circles: water samples from LaBonte and Rock Lake, United States (Steven et al., 2012); Red solid circles: water samples from Qinghai-Tibetan lakes, China (Liu X. et al., 2016); Gold solid circles: samples from this study.

Non-metric dimensional scaling (NMDS) ordinations on the basis of both presence-absence and abundance data showed that benthic (this study) and planktonic (previous studies) algal community compositions were distinctly different (**Figure 6**). Moreover, the algal community composition in American lakes were different from that in Chinese lakes (**Figure 6**). Additionally, phylogenetic analysis indicated that the dominant cyanobacterial OTUs (average relative abundance >0.1%) were mainly affiliated with *Synechococcales* and *Oscillatoriophycideae* (Supplementary Figure S2A) and the relative abundances of those OTUs ranged 0–74.5% (Supplementary Figure S2B) in the studied lakes. Some cyanobacterial OTUs (e.g., OTU98, OTU248, OTU134) occurred in both water and sediment samples, whereas other OTUs only occurred in either water (e.g., OTU80, OTU135) or sediment (e.g., OTU13, OTU14) samples (Supplementary Figure S2B).

## Discussion

It is expected that environmental variables significantly affected the distribution of the benthic algal community in the studied lakes, which was evidenced by the significant correlation between benthic algal community composition and water temperature, light intensity, turbidity, and nutrient-related variables (i.e., DIC, DOC, TP, TN) (**Figure 4**). This finding was in agreement with previous studies on planktonic algal distribution (Riebesell et al., 1993, Riebesell, 2004; Chen and Durbin, 1994; Kenneth, 2002; Elliott et al., 2006; Hare et al., 2007), suggesting that environmental factors could affect the distributions of both planktonic and benthic algal communities. These results were reasonable, because temperature, light intensity and nutrient are crucial factors for algal growth (Geider et al., 1998; Cloern, 1999).

It is remarkable to observe a strong correlation between benthic algal community structures and geographic distance in the studied lakes (**Table 2**), and that the dissimilarities of benthic algal communities increased with increasing geographic distance among the studied lakes (**Figure 5**). This finding is inconsistent with one recent study, which indicated that geographic distance did not significantly affected planktonic algal community structures among lakes (Huang et al., 2014). Such inconsistency may be ascribed to the different studied objects between Huang et al. (2014) and this study (planktonic vs. benthic algal communities). Previous studies have indicated that distinct microbial communities were inhabited in waters (planktonic) and sediments (benthic) (DeLong et al., 1993; Francis et al., 2005; Jiang et al., 2006; Mesbah et al., 2007; Yang et al., 2013), and thus planktonic and benthic microbes may be influenced by different factors among lakes (Yang et al., 2016a). Therefore, it is not surprising to observe the different response of planktonic and benthic algal communities to geographic distance between this and previous studies (Huang et al., 2014). Strong geographic distance effect on benthic algal distribution could be ascribed to the facts that (1) benthic alga were relatively difficult to travel a long distance because they were attached on the benthic sediments and their dispersal might be readily limited by geographic distance; and (2) some environmental variables that might be related to spatial distribution of algal communities were not measured in present study.

It is surprising that salinity did not exhibit significant influence on the benthic algal community structures among the studied lakes (**Figure 4**). Many previous studies have reported that salinity was the strongest limiting factor for microbial distribution in lakes of a large range of salinity (Xing et al., 2009; Liu et al., 2013; Logares et al., 2013; Huang et al., 2014; Liu Y. et al., 2016; Yang et al., 2016b). The studied lakes in present study had a very large salinity range of 0.8–365.6 g/L, and thus salinity was supposed to have strong influence on the benthic algal distribution. Such inconsistency may be ascribed to the following reasons: (1) the impact of water temperature, light and nutrient-related variables (e.g., DIC, DOC, illumination intensity, TN, TP, turbidity) exceeded salinity on the distribution of benthic algal communities, which also was supported by the CCA ordination (**Figure 4**); and (2) micro-niches in the sediments make some attached algal species capable of tolerating a broad salinity range (freshwater to hypersaline), resulting in their insensitivity to salinity change (Oren, 2011, 2015). However, the underlying reasons still await further investigation.

Benthic algal communities in the studied lakes showed different composition from their planktonic counterparts (**Figure 6**), suggesting that the source of some benthic algal taxa could be indigenous. Lake water and sediment are different habitats (having different environmental conditions), and thus they are prone to host distinct microbial communities (DeLong et al., 1993; Jiang et al., 2006; Mesbah et al., 2007; Yang et al., 2013). However, it cannot still be excluded that some benthic algal taxa were derived from upper water column, because we indeed observed the occurrence of some cyanobacterial OTUs in both lake waters and surface sediments (Supplementary Figure S2). Such common algal taxa may be generalists that can utilize a wide spectrum of substrates and thus easily adapt to another new habitat (Hambright et al., 2015). In addition, the distinct algal diversity difference between Chinese and American lakes (**Figure 6**) suggested that geographic isolation may play an important role in influencing algal distribution in lakes. Such geographic isolation effect on microbial distribution has already been reported in many previous studies (Papke et al., 2003; Whitaker et al., 2003; Valverde et al., 2012). However, further investigation is needed to validate the geographic isolation effect on algal distribution observed in this study.

In summary, water temperature, light and nutrient-related variables were more important than salinity in influencing the community compositions of the benthic algae in the studied lakes, and geographic distance could also play an important role in influencing the distribution of benthic algal community. The source of benthic algal taxa in lakes could be partially indigenous or derived from upper water column. The results of this study gave insights into the influence of environmental and spatial factors on the benthic algal distribution in alpine lakes.

## Author Contributions

JY and HJ conceived and designed the experiments. JY, WL, and BW performed the experiments. JY analyzed the data. All authors assisted in writing the manuscript, discussed the results, and commented on the manuscript.

## Conflict of Interest Statement

The authors declare that the research was conducted in the absence of any commercial or financial relationships that could be construed as a potential conflict of interest.

## Footnotes

1 http://blast.ncbi.nlm.nih.gov

2 http://folk.uio.no/ohammer/past/

3 www.ncbi.nlm.nih.gov/blast
