# Supplementary material for: Benthic Algal Community Structures and Their Response to Geographic Distance and Environmental Variables in the Qinghai-Tibetan Lakes With Different Salinity
Source: Front Microbiol. 2018 Mar 27;9:578. doi: 10.3389/fmicb.2018.00578 (PMC5880929; doi:10.3389/fmicb.2018.00578)
Supplement: Supplementary file 1 [file Data_Sheet_1.DOCX]

**Table S1** Taxonomy of the representative OTUs blasted against the NCBI database in this study.

| OTU_ID | Closest strain | Accession number | Similarity (%) | Taxonomy |
| --- | --- | --- | --- | --- |
| OTU308 | *Ankistrodesmus stipitatus* | L42984 | 97 | *Eukaryota; Viridiplantae; Chlorophyta; Chlorophyceae; Sphaeropleales; Selenastraceae; Ankistrodesmus* |
| OTU222 | *Aureoumbra lagunensis* | GQ231542 | 95 | *Eukaryota; Stramenopiles; Pelagophyceae; Pelagomonadales; Aureoumbra* |
| OTU561 | *Aureoumbra lagunensis* | GQ231542 | 97 | *Eukaryota; Stramenopiles; Pelagophyceae; Pelagomonadales; Aureoumbra* |
| OTU527 | *Botryococcus braunii* | LT545991 | 96 | *Eukaryota; Viridiplantae; Chlorophyta; Trebouxiophyceae; Trebouxiophyceae incertae sedis; Botryococcaceae; Botryococcus* |
| OTU239 | *Chara braunii* | EF426577 | 97 | *Eukaryota; Viridiplantae; Streptophyta; Charophyceae; Charales; Characeae; Chara* |
| OTU29 | *Chara braunii* | EF426577 | 100 | *Eukaryota; Viridiplantae; Streptophyta; Charophyceae; Charales; Characeae; Chara* |
| OTU394 | *Chlamydomonas komma* | KP259882 | 97 | *Eukaryota; Viridiplantae; Chlorophyta; Chlorophyceae; Chlamydomonadales; Chlamydomonadaceae; Chlamydomonas* |
| OTU385 | *Chlorella vulgaris* | GU939612 | 99 | *Eukaryota; Viridiplantae; Chlorophyta; Trebouxiophyceae; Chlorellales; Chlorellaceae; Chlorella* |
| OTU152 | *Chlamydomonas pseudopertusa* | L43355 | 96 | *Eukaryota; Viridiplantae; Chlorophyta; Chlorophyceae; Chlamydomonadales; Chlamydomonadaceae; Dangeardinia* |
| OTU350 | *Chlorogonium capillatum* | KT625090 | 99 | *Eukaryota; Viridiplantae; Chlorophyta; Chlorophyceae; Chlamydomonadales; Haematococcaceae; Chlorogonium* |
| OTU276 | *Chloromonas perforata* | KT625416 | 99 | *Eukaryota; Viridiplantae; Chlorophyta; Chlorophyceae; Chlamydomonadales; Chlamydomonadaceae; Chloromonas* |
| OTU379 | *Choricystis parasitica* | KM462878 | 98 | *Eukaryota; Viridiplantae; Chlorophyta; Trebouxiophyceae; Trebouxiophyceae incertae sedis; Choricystis* |
| OTU553 | *Chromera velia* | HM222967 | 90 | *Eukaryota; Alveolata; Chromerida; Chromera* |
| OTU295 | *Chromochloris zofingiensis* | KT199251 | 95 | *Eukaryota; Viridiplantae; Chlorophyta; Chlorophyceae; Sphaeropleales; Chromochloridaceae; Chromochloris* |
| OTU115 | *Chromulina sp.* | KM590766 | 95 | *Eukaryota; Stramenopiles; Chrysophyceae; Chromulinales; Chromulinaceae; Chromulina* |
| OTU245 | *Ankistrodesmus sp.* | KM676721 | 96 | *Eukaryota; Viridiplantae; Chlorophyta; Chlorophyceae; Sphaeropleales; Selenastraceae; Ankistrodesmus* |
| OTU45 | *Dunaliella salina* | KX530454 | 98 | *Eukaryota; Viridiplantae; Chlorophyta; Chlorophyceae; Chlamydomonadales; Dunaliellaceae; Dunaliella* |
| OTU210 | *Neosiphonia japonica* | KC782888 | 97 | *Eukaryota; Rhodophyta; Florideophyceae; Ceramiales; Rhodomelaceae; Neosiphonia* |
| OTU285 | *Neosiphonia japonica* | KC782888 | 97 | *Eukaryota; Rhodophyta; Florideophyceae; Ceramiales; Rhodomelaceae; Neosiphonia* |
| OTU3 | *Neosiphonia japonica* | KC782888 | 99 | *Eukaryota; Rhodophyta; Florideophyceae; Ceramiales; Rhodomelaceae; Neosiphonia* |
| OTU309 | *Euglena carterae* | KT305131 | 94 | *Eukaryota; Euglenozoa; Euglenida; Euglenales; Euglenaceae; Euglena* |
| OTU440 | *Euglena carterae* | KT305131 | 96 | *Eukaryota; Euglenozoa; Euglenida; Euglenales; Euglenaceae; Euglena* |
| OTU444 | *Euglena carterae* | KT305131 | 96 | *Eukaryota; Euglenozoa; Euglenida; Euglenales; Euglenaceae; Euglena* |
| OTU59 | *Euglena carterae* | KT305131 | 98 | *Eukaryota; Euglenozoa; Euglenida; Euglenales; Euglenaceae; Euglena* |
| OTU425 | *Euglenaria anabaena* | KT305149 | 97 | *Eukaryota; Euglenozoa; Euglenida; Euglenales; Euglenaceae; Euglenaria* |
| OTU114 | *Eutreptia viridis* | JN643723 | 99 | *Eukaryota; Euglenozoa; Euglenida; Eutreptiales; Eutreptia* |
| OTU208 | *Eutreptia viridis* | JN643723 | 97 | *Eukaryota; Euglenozoa; Euglenida; Eutreptiales; Eutreptia* |
| OTU460 | *Synura uvella* | KM590761 | 90 | *Eukaryota; Stramenopiles; Synurophyceae; Synurales* |
| OTU203 | *Kryptoperidinium foliaceum* | GU591328 | 98 | *Eukaryota; Alveolata; Dinophyceae; Peridiniales; Peridiniaceae; Kryptoperidinium* |
| OTU241 | *Kryptoperidinium foliaceum* | GU591328 | 98 | *Eukaryota; Alveolata; Dinophyceae; Peridiniales; Peridiniaceae; Kryptoperidinium* |
| OTU168 | *Mallomonas corymbosa* | KM817948 | 93 | *Eukaryota; Stramenopiles; Synurophyceae; Synurales; Mallomonadaceae; Mallomonas* |
| OTU106 | *Chlamydomonas pitschmannii* | Z15152 | 93 | *Eukaryota; Viridiplantae; Chlorophyta; Chlorophyceae; Chlamydomonadales; Chlamydomonadaceae; Chlamydomonas* |
| OTU315 | *Chlamydomonas geitleri* | X68892 | 98 | *Eukaryota; Viridiplantae; Chlorophyta; Chlorophyceae; Chlamydomonadales; Chlamydomonadaceae; Chlamydomonas* |
| OTU433 | *Chlamydomonas leiostraca* | KX828176 | 95 | *Eukaryota; Viridiplantae; Chlorophyta; Chlorophyceae; Chlamydomonadales; Chlamydomonadaceae; Chlamydomonas* |
| OTU255 | *Mychonastes homosphaera* | KT199249 | 99 | *Eukaryota; Viridiplantae; Chlorophyta; Chlorophyceae; Sphaeropleales; Mychonastaceae; Mychonastes* |
| OTU381 | *Vischeria* sp. | KX839261 | 98 | *Eukaryota; Stramenopiles; Eustigmatophyceae; Eustigmatales; Eustigmataceae; Vischeria.* |
| OTU147 | *Nannochloropsis salina* | KJ410685 | 100 | *Eukaryota; Stramenopiles; Eustigmatophyceae; Eustigmatales; Monodopsidaceae; Nannochloropsis* |
| OTU572 | *Neochloris aquatica* | KT199248 | 97 | *Eukaryota; Viridiplantae; Chlorophyta; Chlorophyceae; Sphaeropleales; Neochloridaceae; Neochloris* |
| OTU347 | *Ochromonas danica* | KM590767 | 92 | *Eukaryota; Stramenopiles; Chrysophyceae; Chromulinales; Chromulinaceae; Ochromonas* |
| OTU256 | *Ochromonas* sp. | KJ877675 | 96 | *Eukaryota; Stramenopiles; Chrysophyceae; Chromulinales; Chromulinaceae; Ochromonas* |
| OTU337 | *Ochromonas* sp. | KJ877675 | 92 | *Eukaryota; Stramenopiles; Chrysophyceae; Chromulinales; Chromulinaceae; Ochromonas* |
| OTU538 | *Ochromonas* sp.*.* | KJ877675 | 90 | *Eukaryota; Stramenopiles; Chrysophyceae; Chromulinales; Chromulinaceae; Ochromonas* |
| OTU164 | *Pavlova lutheri* | KC573041 | 96 | *Eukaryota; Haptophyceae; Pavlovales; Pavlovaceae; Pavlova* |
| OTU434 | *Pavlova lutheri* | KC573041 | 97 | *Eukaryota; Haptophyceae; Pavlovales; Pavlovaceae; Pavlova* |
| OTU463 | *Pavlova lutheri* | KC573041 | 98 | *Eukaryota; Haptophyceae; Pavlovales; Pavlovaceae; Pavlova* |
| OTU264 | *Oocystis solitaria* | FJ968739 | 94 | *Eukaryota; Viridiplantae; Chlorophyta; Trebouxiophyceae; Chlorellales; Oocystaceae; Oocystis* |
| OTU18 | *Pseudo-nitzschia multiseries* | KR709240 | 98 | *Eukaryota; Stramenopiles; Bacillariophyta; Bacillariophyceae; Bacillariophycidae; Bacillariales; Bacillariaceae; Pseudo-nitzschia* |
| OTU205 | *Pyramimonas disomata* | FN563101 | 98 | *Eukaryota; Viridiplantae; Chlorophyta; prasinophytes; Pyramimonadales; Pyramimonas* |
| OTU110 | *Rhodomonas lens* | KP142647 | 99 | *Eukaryota; Cryptophyta; Pyrenomonadales; Pyrenomonadaceae; Rhodomonas* |
| OTU367 | *Scenedesmus* sp. | GU939615 | 99 | *Eukaryota; Viridiplantae; Chlorophyta; Chlorophyceae; Sphaeropleales; Scenedesmaceae; Scenedesmus* |
| OTU331 | *Scenedesmus* sp. | HE717103 | 91 | *Eukaryota; Viridiplantae; Chlorophyta; Chlorophyceae; Sphaeropleales; Scenedesmaceae; Scenedesmus* |
| OTU306 | *Scenedesmus* sp. | KF805721 | 99 | *Eukaryota; Viridiplantae; Chlorophyta; Chlorophyceae; Sphaeropleales; Scenedesmaceae; Scenedesmus* |
| OTU226 | *Tetraselmis cordiformis* | HE610165 | 100 | *Eukaryota; Viridiplantae; Chlorophyta; Chlorodendrophyceae; Chlorodendrales; Chlorodendraceae; Tetraselmis* |
| OTU218 | *Tetraselmis marina* | HE610166 | 94 | *Eukaryota; Viridiplantae; Chlorophyta; Chlorodendrophyceae; Chlorodendrales; Chlorodendraceae; Tetraselmis* |
| OTU174 | *Peridiniopsis penardii* | JQ639746 | 99 | *Eukaryota; Alveolata; Dinophyceae; Peridiniales; Glenodiniaceae; Peridiniopsis* |
| OTU372 | *Trachydiscus minutus* | KJ624065 | 99 | *Eukaryota; Stramenopiles; Eustigmatophyceae; Goniochloridales; Trachydiscus* |
| OTU38 | *Tribonema affine* | KM676797 | 98 | *Eukaryota; Stramenopiles; PX clade; Xanthophyceae; Tribonematales; Tribonemataceae; Tribonema* |
| OTU260 | *Scenedesmus* sp. | GU939614 | 98 | *Eukaryota; Viridiplantae; Chlorophyta; Chlorophyceae; Sphaeropleales; Scenedesmaceae; Scenedesmus* |
| OTU384 | *Eunotia naegelii* | KF733443 | 97 | *Eukaryota; Stramenopiles; Bacillariophyta; Bacillariophyceae; Eunotiophycidae; Eunotiales; Eunotiaceae; Eunotia* |
| OTU61 | *Emiliania huxleyi* | JN022705 | 97 | *Eukaryota; Haptophyceae; Isochrysidales; Noelaerhabdaceae; Emiliania* |
| OTU240 | *Micromonas pusilla* | FN563097 | 99 | *Eukaryota; Viridiplantae; Chlorophyta; prasinophytes; Mamiellophyceae; Mamiellales; Mamiellaceae; Micromonas* |
| OTU259 | *Eunotia naegelii* | KF733443 | 93 | *Eukaryota; Stramenopiles; Bacillariophyta; Bacillariophyceae; Eunotiophycidae; Eunotiales; Eunotiaceae; Eunotia* |
| OTU525 | *Cerataulina daemon* | KJ958484 | 88 | *Eukaryota; Stramenopiles; Bacillariophyta; Mediophyceae; Biddulphiophycidae; Hemiaulales; Hemiaulaceae; Cerataulina* |
| OTU446 | *Fistulifera solaris* | AP011960 | 97 | *Eukaryota; Stramenopiles; Bacillariophyta; Bacillariophyceae; Bacillariophycidae; Naviculales; Naviculaceae; Fistulifera* |
| OTU101 | *Fistulifera solaris* | AP011960 | 98 | *Eukaryota; Stramenopiles; Bacillariophyta; Bacillariophyceae; Bacillariophycidae; Naviculales; Naviculaceae; Fistulifera* |
| OTU142 | *Marvania geminata* | KM462888 | 97 | *Eukaryota; Viridiplantae; Chlorophyta; Trebouxiophyceae; Chlorellales; Chlorellaceae; Marvania* |
| OTU184 | *Neosiphonia japonica* | KC782888 | 95 | *Eukaryota; Rhodophyta; Florideophyceae; Ceramiales; Rhodomelaceae; Neosiphonia* |
| OTU219 | *Monodopsis* sp. | KX839260 | 92 | *Eukaryota; Stramenopiles; Eustigmatophyceae; Eustigmatales; Monodopsidaceae; Monodopsis* |
| OTU293 | *Bacillariophyceae* sp. | KM676741 | 95 | *Eukaryota; Stramenopiles; Bacillariophyta; Bacillariophyceae; unclassified Bacillariophyceae* |
| OTU311 | *Tribonema affine* | KM676797 | 91 | *Eukaryota; Stramenopiles; PX clade; Xanthophyceae; Tribonematales; Tribonemataceae; Tribonema* |
| OTU42 | *Vaucheria* sp. | KM676569 | 96 | *Eukaryota; Stramenopiles; PX clade; Xanthophyceae; Vaucheriales; Vaucheriaceae; Vaucheria* |
| OTU47 | *Cerataulina daemon* | KJ958484 | 97 | *Eukaryota; Stramenopiles; Bacillariophyta; Mediophyceae; Biddulphiophycidae; Hemiaulales; Hemiaulaceae; Cerataulina* |
| OTU51 | *Cryptomonas phaseolus* | KF907430 | 89 | *Eukaryota; Cryptophyta; Cryptomonadales; Cryptomonadaceae; Cryptomonas* |
| OTU529 | *Choricystis parasitica* | KM462878 | 95 | *Eukaryota; Viridiplantae; Chlorophyta; Trebouxiophyceae; Trebouxiophyceae incertae sedis; Choricystis* |
| OTU562 | *Tribonema affine* | KM676797 | 94 | *Eukaryota; Stramenopiles; PX clade; Xanthophyceae; Tribonematales; Tribonemataceae; Tribonema* |
| OTU7 | *Phaeodactylum tricornutum* | EF067920 | 98 | *Eukaryota; Stramenopiles; Bacillariophyta; Bacillariophyceae; Bacillariophycidae; Naviculales; Phaeodactylaceae; Phaeodactylum* |
| OTU83 | *Neosiphonia japonica* | KC782888 | 97 | *Eukaryota; Rhodophyta; Florideophyceae; Ceramiales; Rhodomelaceae; Neosiphonia* |
| OTU88 | *Geminella minor* | KM462883 | 98 | *Eukaryota; Viridiplantae; Chlorophyta; Geminella* |
| OTU92 | *Lotharella* sp. | KF438023 | 98 | *Eukaryota; Rhizaria; Cercozoa; Chlorarachniophyceae; Lotharella.* |
| OTU192 | *Nostoc* sp. | NR_076095 | 94 | *Bacteria; Cyanobacteria; Nostocales; Nostocaceae; Nostoc.* |
| OTU15 | *Leptolyngbya fragilis* | KR676350 | 99 | *Bacteria; Cyanobacteria; Synechococcales; Leptolyngbyaceae; Leptolyngbya* |
| OTU473 | *Leptolyngbya fragilis* | KR676349 | 93 | *Bacteria; Cyanobacteria; Synechococcales; Leptolyngbyaceae; Leptolyngbya* |
| OTU139 | *Leptolyngbya fragilis* | KR676350 | 92 | *Bacteria; Cyanobacteria; Synechococcales; Leptolyngbyaceae; Leptolyngbya* |
| OTU78 | *Leptolyngbya fragilis* | KR676350 | 92 | *Bacteria; Cyanobacteria; Synechococcales; Leptolyngbyaceae; Leptolyngbya* |
| OTU330 | *Leptolyngbya* sp. | KC848642 | 95 | *Bacteria; Cyanobacteria; Synechococcales; Leptolyngbyaceae; Leptolyngbya* |
| OTU102 | *Leptolyngbya* sp. | KC848642 | 93 | *Bacteria; Cyanobacteria; Synechococcales; Leptolyngbyaceae; Leptolyngbya* |
| OTU135 | *Synechococcus lividus* | DQ421379 | 93 | *Bacteria; Cyanobacteria; Synechococcales; Synechococcaceae; Synechococcus* |
| OTU170 | *Nodosilinea* sp. | KX859297 | 94 | *Bacteria; Cyanobacteria; Synechococcales; Leptolyngbyaceae; Nodosilinea* |
| OTU220 | *Leptolyngbya* sp. | KC848642 | 91 | *Bacteria; Cyanobacteria; Synechococcales; Leptolyngbyaceae; Leptolyngbya* |
| OTU144 | *Leptolyngbya boryana* | AY584526 | 92 | *Bacteria; Cyanobacteria; Synechococcales; Leptolyngbyaceae; Leptolyngbya* |
| OTU182 | *Plectonema terebrans* | AY584525 | 92 | *Bacteria; Cyanobacteria; Oscillatoriophycideae; Oscillatoriales; Oscillatoriaceae; Plectonema* |
| OTU36 | *Plectonema terebrans* | AY584525 | 93 | *Bacteria; Cyanobacteria; Oscillatoriophycideae; Oscillatoriales; Oscillatoriaceae; Plectonema* |
| OTU495 | *Plectonema terebrans* | AY584525 | 90 | *Bacteria; Cyanobacteria; Oscillatoriophycideae; Oscillatoriales; Oscillatoriaceae; Plectonema* |
| OTU5 | *Plectonema terebrans* | AY584525 | 94 | *Bacteria; Cyanobacteria; Oscillatoriophycideae; Oscillatoriales; Oscillatoriaceae; Plectonema* |
| OTU580 | *Synechococcus* sp. | CP006270 | 90 | *Bacteria; Cyanobacteria; Synechococcales; Synechococcaceae; Synechococcus* |
| OTU193 | *Acaryochloris marina* | NR_076538 | 91 | *Bacteria; Cyanobacteria; Synechococcales; Acaryochloridaceae; Acaryochloris* |
| OTU359 | *Cyanobacterium endosymbiont* | AP012549 | 99 | *Bacteria; Cyanobacteria* |
| OTU492 | *Cyanobacterium aponinum* | NR_102522 | 93 | *Bacteria; Cyanobacteria; Oscillatoriophycideae; Chroococcales; Cyanobacteriaceae; Cyanobacterium* |
| OTU313 | *Cyanobacterium stanieri* | NR_102523 | 97 | *Bacteria; Cyanobacteria; Oscillatoriophycideae; Chroococcales; Cyanobacteriaceae; Cyanobacterium* |
| OTU71 | *Cyanobacterium stanieri* | NR_102523 | 97 | *Bacteria; Cyanobacteria; Oscillatoriophycideae; Chroococcales; Cyanobacteriaceae; Cyanobacterium* |
| OTU80 | *Cyanobacterium stanieri* | NR_102523 | 97 | *Bacteria; Cyanobacteria; Oscillatoriophycideae; Chroococcales; Cyanobacteriaceae; Cyanobacterium* |
| OTU286 | *Cyanobium* sp. | LT578417 | 97 | *Bacteria; Cyanobacteria; Synechococcales; Synechococcaceae; Cyanobium* |
| OTU11 | *Synechococcus* sp. | AM709628 | 100 | *Bacteria; Cyanobacteria; Synechococcales; Synechococcaceae; Cyanobium* |
| OTU60 | *Synechococcus* sp. | CP018091 | 98 | *Bacteria; Cyanobacteria; Synechococcales; Synechococcaceae; Synechococcus* |
| OTU72 | *Synechococcus* sp. | CP018091 | 98 | *Bacteria; Cyanobacteria; Synechococcales; Synechococcaceae; Synechococcus* |
| OTU105 | *Synechococcus* sp. | AM709627 | 94 | *Bacteria; Cyanobacteria; Synechococcales; Synechococcaceae; Synechococcus* |
| OTU130 | *Synechococcus* sp. | AM709627 | 93 | *Bacteria; Cyanobacteria; Synechococcales; Synechococcaceae; Synechococcus* |
| OTU4 | *Synechococcus* sp. | AM709627 | 99 | *Bacteria; Cyanobacteria; Synechococcales; Synechococcaceae; Synechococcus* |
| OTU416 | *Synechococcus* sp. | AM709627 | 99 | *Bacteria; Cyanobacteria; Synechococcales; Synechococcaceae; Synechococcus* |
| OTU87 | *Synechococcus* sp. | AM709627 | 97 | *Bacteria; Cyanobacteria; Synechococcales; Synechococcaceae; Synechococcus* |
| OTU1 | *Synechococcus* sp. | AM709627 | 96 | *Bacteria; Cyanobacteria; Synechococcales; Synechococcaceae; Synechococcus* |
| OTU127 | *Synechococcus* sp.*.* | AM709627 | 92 | *Bacteria; Cyanobacteria; Synechococcales; Synechococcaceae; Synechococcus* |
| OTU143 | *Synechococcus* sp. | AM709627 | 90 | *Bacteria; Cyanobacteria; Synechococcales; Synechococcaceae; Synechococcus* |
| OTU176 | *Synechococcus* sp. | AM709627 | 93 | *Bacteria; Cyanobacteria; Synechococcales; Synechococcaceae; Synechococcus* |
| OTU216 | *Synechococcus* sp. | AM709627 | 91 | *Bacteria; Cyanobacteria; Synechococcales; Synechococcaceae; Synechococcus* |
| OTU217 | *Synechococcus* sp. | AM709627 | 92 | *Bacteria; Cyanobacteria; Synechococcales; Synechococcaceae; Synechococcus* |
| OTU242 | *Synechococcus* sp. | AM709627 | 93 | *Bacteria; Cyanobacteria; Synechococcales; Synechococcaceae; Synechococcus* |
| OTU477 | *Synechococcus* sp. | AM709627 | 88 | *Bacteria; Cyanobacteria; Synechococcales; Synechococcaceae; Synechococcus* |
| OTU524 | *Synechococcus* sp. | AM709627 | 93 | *Bacteria; Cyanobacteria; Synechococcales; Synechococcaceae; Synechococcus* |
| OTU526 | *Synechococcus* sp. | AM709627 | 91 | *Bacteria; Cyanobacteria; Synechococcales; Synechococcaceae; Synechococcus* |
| OTU535 | *Synechococcus* sp. | AM709627 | 92 | *Bacteria; Cyanobacteria; Synechococcales; Synechococcaceae; Synechococcus* |
| OTU550 | *Synechococcus* sp. | AM709627 | 93 | *Bacteria; Cyanobacteria; Synechococcales; Synechococcaceae; Synechococcus* |
| OTU107 | *Synechococcus* sp. | AM709627 | 95 | *Bacteria; Cyanobacteria; Synechococcales; Synechococcaceae; Synechococcus* |
| OTU272 | *Oscillatoria* sp. | AY584514 | 90 | *Bacteria; Cyanobacteria; Oscillatoriophycideae; Oscillatoriales; Oscillatoriaceae; Oscillatoria* |
| OTU325 | *Synechococcus* sp.*.* | AM709627 | 96 | *Bacteria; Cyanobacteria; Synechococcales; Synechococcaceae; Synechococcus* |
| OTU62 | *Synechococcus* sp. | CP018091 | 96 | *Bacteria; Cyanobacteria; Synechococcales; Synechococcaceae; Synechococcus* |
| OTU28 | *Cyanothece* sp. | NR_076532 | 92 | *Bacteria; Cyanobacteria; Oscillatoriophycideae; Oscillatoriales; Cyanothecaceae; Cyanothece* |
| OTU398 | *Spirulina* sp. | AM709631 | 90 | *Bacteria; Cyanobacteria; Spirulinales; Spirulinaceae; Spirulina* |
| OTU22 | *Cyanothece* sp.*.* | CP001344 | 93 | *Bacteria; Cyanobacteria; Oscillatoriophycideae; Oscillatoriales; Cyanothecaceae; Cyanothece* |
| OTU481 | *Cyanothece* sp. | CP001344 | 91 | *Bacteria; Cyanobacteria; Oscillatoriophycideae; Oscillatoriales; Cyanothecaceae; Cyanothece* |
| OTU50 | *Cyanothece* sp. | CP001344 | 94 | *Bacteria; Cyanobacteria; Oscillatoriophycideae; Oscillatoriales; Cyanothecaceae; Cyanothece* |
| OTU70 | *Cyanothece* sp. | CP001344 | 93 | *Bacteria; Cyanobacteria; Oscillatoriophycideae; Oscillatoriales; Cyanothecaceae; Cyanothece* |
| OTU214 | *Cyanothece* sp. | NR_076616 | 95 | *Bacteria; Cyanobacteria; Oscillatoriophycideae; Oscillatoriales; Cyanothecaceae; Cyanothece* |
| OTU270 | *Pleurocapsa* sp. | NR_102536 | 92 | *Bacteria; Cyanobacteria; Pleurocapsales; Hyellaceae; Pleurocapsa.* |
| OTU363 | *Pleurocapsa* sp. | NR_102536 | 92 | *Bacteria; Cyanobacteria; Pleurocapsales; Hyellaceae; Pleurocapsa.* |
| OTU378 | *Pleurocapsa* sp. | NR_102536 | 92 | *Bacteria; Cyanobacteria; Pleurocapsales; Hyellaceae; Pleurocapsa.* |
| OTU404 | *Pleurocapsa* sp. | NR_102536 | 91 | *Bacteria; Cyanobacteria; Pleurocapsales; Hyellaceae; Pleurocapsa.* |
| OTU414 | *Pleurocapsa* sp. | NR_102536 | 91 | *Bacteria; Cyanobacteria; Pleurocapsales; Hyellaceae; Pleurocapsa.* |
| OTU521 | *Pleurocapsa* sp. | NR_102536 | 91 | *Bacteria; Cyanobacteria; Pleurocapsales; Hyellaceae; Pleurocapsa.* |
| OTU186 | *Cyanothece* sp. | CP001344 | 90 | *Bacteria; Cyanobacteria; Oscillatoriophycideae; Oscillatoriales; Cyanothecaceae; Cyanothece* |
| OTU19 | *Geminocystis* sp. | AP014815 | 97 | *Bacteria; Cyanobacteria; Oscillatoriophycideae; Chroococcales; Chroococcaceae; Geminocystis* |
| OTU369 | *Synechocystis* sp. | CP012832 | 88 | *Bacteria; Cyanobacteria; Synechococcales; Merismopediaceae; Synechocystis* |
| OTU231 | *Geminocystis* sp. | AP014821 | 96 | *Bacteria; Cyanobacteria; Oscillatoriophycideae; Chroococcales; Chroococcaceae; Geminocystis* |
| OTU198 | *Cyanobacterium aponinum* | NR_102522 | 94 | *Bacteria; Cyanobacteria; Oscillatoriophycideae; Chroococcales; Cyanobacteriaceae; Cyanobacterium* |
| OTU365 | *Gloeocapsa* sp. | NR_102540 | 98 | *Bacteria; Cyanobacteria; Oscillatoriophycideae; Chroococcales; Chroococcaceae; Gloeocapsa* |
| OTU456 | *Hyella* sp. | KR676351 | 92 | *Bacteria; Cyanobacteria; Pleurocapsales; Hyellaceae; Hyella* |
| OTU84 | *Hyella* sp. | KR676351 | 91 | *Bacteria; Cyanobacteria; Pleurocapsales; Hyellaceae; Hyella* |
| OTU94 | *Hyella* sp. | KR676351 | 91 | *Bacteria; Cyanobacteria; Pleurocapsales; Hyellaceae; Hyella* |
| OTU577 | *Cyanothece* sp. | CP001344 | 89 | *Bacteria; Cyanobacteria; Oscillatoriophycideae; Oscillatoriales; Cyanothecaceae; Cyanothece* |
| OTU138 | *Hyella* sp. | KR676351 | 92 | *Bacteria; Cyanobacteria; Pleurocapsales; Hyellaceae; Hyella* |
| OTU124 | *Synechococcus* sp. | AP017959 | 88 | *Bacteria; Cyanobacteria; Synechococcales; Synechococcaceae; Synechococcus* |
| OTU200 | *Pleurocapsa* sp. | KC848633 | 95 | *Bacteria; Cyanobacteria; Pleurocapsales; Hyellaceae; Pleurocapsa* |
| OTU53 | *Leptolyngbya* sp. | KC848649 | 87 | *Bacteria; Cyanobacteria; Synechococcales; Leptolyngbyaceae; Leptolyngbya* |
| OTU503 | *Stanieria cyanosphaera* | NR_102529 | 94 | *Bacteria; Cyanobacteria; Pleurocapsales; Dermocarpellaceae; Stanieria* |
| OTU327 | *Synechococcus* sp. | CT978603 | 95 | *Bacteria; Cyanobacteria; Synechococcales; Synechococcaceae; Synechococcus* |
| OTU513 | *Synechococcus lividus* | DQ421379 | 93 | *Bacteria; Cyanobacteria; Synechococcales; Synechococcaceae; Synechococcus* |
| OTU54 | *Synechococcus* sp. | CP000240 | 94 | *Bacteria; Cyanobacteria; Synechococcales; Synechococcaceae; Synechococcus* |
| OTU90 | *Synechococcus* sp. | CP003558 | 90 | *Bacteria; Cyanobacteria; Synechococcales; Synechococcaceae; Synechococcus* |
| OTU316 | *Geitlerinema* sp. | NR_102539 | 91 | *Bacteria; Cyanobacteria; Oscillatoriophycideae; Oscillatoriales; Coleofasciculaceae; Geitlerinema* |
| OTU528 | *Leptolyngbya* sp. | KC848649 | 91 | *Bacteria; Cyanobacteria; Synechococcales; Leptolyngbyaceae; Leptolyngbya* |
| OTU99 | *Plectonema terebrans* | AY584525 | 91 | *Bacteria; Cyanobacteria; Oscillatoriophycideae; Oscillatoriales; Oscillatoriaceae; Plectonema* |
| OTU177 | *Synechocystis* sp. | CP012832 | 96 | *Bacteria; Cyanobacteria; Synechococcales; Merismopediaceae; Synechocystis* |
| OTU466 | *Synechocystis* sp. | CP012832 | 96 | *Bacteria; Cyanobacteria; Synechococcales; Merismopediaceae; Synechocystis* |
| OTU75 | *Synechocystis* sp. | CP012832 | 100 | *Bacteria; Cyanobacteria; Synechococcales; Merismopediaceae; Synechocystis* |
| OTU82 | *Synechocystis* sp. | CP012832 | 94 | *Bacteria; Cyanobacteria; Synechococcales; Merismopediaceae; Synechocystis* |
| OTU471 | *Synechococcus* sp. | CP006471 | 93 | *Bacteria; Cyanobacteria; Synechococcales; Synechococcaceae; Synechococcus* |
| OTU530 | *Synechocystis* sp. | CP012832 | 92 | *Bacteria; Cyanobacteria; Synechococcales; Merismopediaceae; Synechocystis* |
| OTU362 | *Gloeocapsa* sp. | NR_102540 | 91 | *Bacteria; Cyanobacteria; Oscillatoriophycideae; Chroococcales; Chroococcaceae; Gloeocapsa* |
| OTU37 | *Gloeocapsa* sp. | NR_102540 | 91 | *Bacteria; Cyanobacteria; Oscillatoriophycideae; Chroococcales; Chroococcaceae; Gloeocapsa* |
| OTU12 | *Chamaesiphon minutus* | NR_102544 | 91 | *Bacteria; Cyanobacteria; Synechococcales; Chamaesiphonaceae; Chamaesiphon* |
| OTU41 | *Chamaesiphon minutus* | NR_102544 | 98 | *Bacteria; Cyanobacteria; Synechococcales; Chamaesiphonaceae; Chamaesiphon* |
| OTU545 | *Chamaesiphon minutus* | NR_102544 | 91 | *Bacteria; Cyanobacteria; Synechococcales; Chamaesiphonaceae; Chamaesiphon* |
| OTU66 | *Chamaesiphon minutus* | NR_102544 | 99 | *Bacteria; Cyanobacteria; Synechococcales; Chamaesiphonaceae; Chamaesiphon* |
| OTU537 | *Crinalium epipsammum* | CP003620 | 99 | *Bacteria; Cyanobacteria; Oscillatoriophycideae; Oscillatoriales; Gomontiellaceae; Crinalium* |
| OTU58 | *Crinalium epipsammum* | CP003620 | 92 | *Bacteria; Cyanobacteria; Oscillatoriophycideae; Oscillatoriales; Gomontiellaceae; Crinalium* |
| OTU294 | *Pleurocapsa* sp. | NR_102536 | 92 | *Bacteria; Cyanobacteria; Pleurocapsales; Hyellaceae; Pleurocapsa.* |
| OTU96 | *Halothece* sp. | NR_102528 | 97 | *Bacteria; Cyanobacteria; Oscillatoriophycideae; Chroococcales; Aphanothecaceae; Halothece cluster; Halothece* |
| OTU441 | *Leptolyngbya boryana* | JN088474 | 94 | *Bacteria; Cyanobacteria; Synechococcales; Leptolyngbyaceae; Leptolyngbya* |
| OTU284 | *Leptolyngbya* sp. | KC848643 | 95 | *Bacteria; Cyanobacteria; Synechococcales; Leptolyngbyaceae; Leptolyngbya* |
| OTU204 | *Leptolyngbya* sp. | AP017367 | 92 | *Bacteria; Cyanobacteria; Synechococcales; Leptolyngbyaceae; Leptolyngbya* |
| OTU196 | *Leptolyngbya* sp.*.* | KC848649 | 94 | *Bacteria; Cyanobacteria; Synechococcales; Leptolyngbyaceae; Leptolyngbya* |
| OTU224 | *Leptolyngbya* sp. | KC848649 | 91 | *Bacteria; Cyanobacteria; Synechococcales; Leptolyngbyaceae; Leptolyngbya* |
| OTU202 | *Geitlerinema* sp. | NR_102539 | 91 | *Bacteria; Cyanobacteria; Oscillatoriophycideae; Oscillatoriales; Coleofasciculaceae; Geitlerinema* |
| OTU16 | *Leptolyngbya* sp. | KC848647 | 93 | *Bacteria; Cyanobacteria; Synechococcales; Leptolyngbyaceae; Leptolyngbya* |
| OTU183 | *Oscillatoria* sp. | AY584515 | 99 | *Bacteria; Cyanobacteria; Oscillatoriophycideae; Oscillatoriales; Oscillatoriaceae; Oscillatoria* |
| OTU336 | *Microcoleus chthonoplastes* | AM709630 | 99 | *Bacteria; Cyanobacteria; Oscillatoriophycideae; Oscillatoriales; Coleofasciculaceae; Coleofasciculus* |
| OTU199 | *Cyanobacterium* | KM676779 | 99 | *Bacteria; Cyanobacteria* |
| OTU178 | *Leptolyngbya* sp*.* | AY584521 | 92 | *Bacteria; Cyanobacteria; Synechococcales; Leptolyngbyaceae; Nodosilinea* |
| OTU418 | *Nodosilinea* sp. | KX859297 | 91 | *Bacteria; Cyanobacteria; Synechococcales; Leptolyngbyaceae; Nodosilinea* |
| OTU510 | *Nodosilinea* sp. | KX859297 | 91 | *Bacteria; Cyanobacteria; Synechococcales; Leptolyngbyaceae; Nodosilinea* |
| OTU169 | *Nodosilinea* sp. | KC848652 | 95 | *Bacteria; Cyanobacteria; Synechococcales; Leptolyngbyaceae; Nodosilinea* |
| OTU251 | *Nodosilinea* sp. | KC848652 | 98 | *Bacteria; Cyanobacteria; Synechococcales; Leptolyngbyaceae; Nodosilinea* |
| OTU531 | *Nodosilinea* sp. | KC848652 | 95 | *Bacteria; Cyanobacteria; Synechococcales; Leptolyngbyaceae; Nodosilinea* |
| OTU155 | *Nodosilinea epilithica* | KC848650 | 95 | *Bacteria; Cyanobacteria; Synechococcales; Leptolyngbyaceae; Nodosilinea* |
| OTU2 | *Nodosilinea* sp. | KC848652 | 98 | *Bacteria; Cyanobacteria; Synechococcales; Leptolyngbyaceae; Nodosilinea* |
| OTU380 | *Nodosilinea* sp. | KX859297 | 100 | *Bacteria; Cyanobacteria; Synechococcales; Leptolyngbyaceae; Nodosilinea* |
| OTU39 | *Nodosilinea* sp. | KX859297 | 98 | *Bacteria; Cyanobacteria; Synechococcales; Leptolyngbyaceae; Nodosilinea* |
| OTU89 | *Nodosilinea* sp. | KX859297 | 96 | *Bacteria; Cyanobacteria; Synechococcales; Leptolyngbyaceae; Nodosilinea* |
| OTU43 | *Oscillatoria acuminata* | NR_102542 | 97 | *Bacteria; Cyanobacteria; Oscillatoriophycideae; Oscillatoriales; Oscillatoriaceae; Oscillatoria* |
| OTU206 | *Oscillatoria* sp. | AY584514 | 92 | *Bacteria; Cyanobacteria; Oscillatoriophycideae; Oscillatoriales; Oscillatoriaceae; Oscillatoria* |
| OTU268 | *Oscillatoria* sp. | AY584514 | 93 | *Bacteria; Cyanobacteria; Oscillatoriophycideae; Oscillatoriales; Oscillatoriaceae; Oscillatoria* |
| OTU297 | *Oscillatoria* sp. | AY584514 | 92 | *Bacteria; Cyanobacteria; Oscillatoriophycideae; Oscillatoriales; Oscillatoriaceae; Oscillatoria* |
| OTU34 | *Oscillatoria* sp. | AY584514 | 99 | *Bacteria; Cyanobacteria; Oscillatoriophycideae; Oscillatoriales; Oscillatoriaceae; Oscillatoria* |
| OTU271 | *Planktothrix agardhii* | LO018304 | 99 | *Bacteria; Cyanobacteria; Oscillatoriophycideae; Oscillatoriales; Microcoleaceae; Planktothrix* |
| OTU64 | *Cyanobacterium* | KM676742 | 94 | *Bacteria; Cyanobacteria* |
| OTU91 | *Synechococcus* sp. | CP003594 | 97 | *Bacteria; Cyanobacteria; Synechococcales; Synechococcaceae; Synechococcus* |
| OTU112 | *Cyanobacterium* | KM676720 | 99 | *Bacteria; Cyanobacteria.* |
| OTU180 | *Spirulina* sp. | AM709631 | 92 | *Bacteria; Cyanobacteria; Spirulinales; Spirulinaceae; Spirulina* |
| OTU33 | *Spirulina* sp. | AM709631 | 96 | *Bacteria; Cyanobacteria; Spirulinales; Spirulinaceae; Spirulina* |
| OTU52 | *Spirulina* sp. | AM709631 | 100 | *Bacteria; Cyanobacteria; Spirulinales; Spirulinaceae; Spirulina* |
| OTU77 | *Spirulina* sp. | AM709631 | 92 | *Bacteria; Cyanobacteria; Spirulinales; Spirulinaceae; Spirulina* |
| OTU104 | *Oscillatoria acuminata* | NR_102542 | 91 | *Bacteria; Cyanobacteria; Oscillatoriophycideae; Oscillatoriales; Oscillatoriaceae; Oscillatoria* |
| OTU136 | *Trichodesmium erythraeum* | NR_076432 | 91 | *Bacteria; Cyanobacteria; Oscillatoriophycideae; Oscillatoriales; Microcoleaceae; Trichodesmium* |
| OTU514 | *Leptolyngbya* sp*.* | KC848649 | 93 | *Bacteria; Cyanobacteria; Synechococcales; Leptolyngbyaceae; Leptolyngbya* |
| OTU173 | *Aphanizomenon ovalisporum* | JF768745 | 95 | *Bacteria; Cyanobacteria; Nostocales; Aphanizomenonaceae; Chrysosporum* |
| OTU233 | *Aphanizomenon ovalisporum* | JF768745 | 95 | *Bacteria; Cyanobacteria; Nostocales; Aphanizomenonaceae; Chrysosporum* |
| OTU13 | *Calothrix* sp. | KC848593 | 96 | *Bacteria; Cyanobacteria; Nostocales; Rivulariaceae; Calothrix.* |
| OTU326 | *Calothrix* sp. | KC848593 | 97 | *Bacteria; Cyanobacteria; Nostocales; Rivulariaceae; Calothrix.* |
| OTU123 | *Nodularia harveyana* | AY584513 | 99 | *Bacteria; Cyanobacteria; Nostocales; Aphanizomenonaceae; Nodularia* |
| OTU392 | *Nodularia spumigena* | CP007203 | 98 | *Bacteria; Cyanobacteria; Nostocales; Aphanizomenonaceae; Nodularia* |
| OTU397 | *Nostoc* sp. | NR_076095 | 98 | *Bacteria; Cyanobacteria; Nostocales; Nostocaceae; Nostoc* |
| OTU491 | *Rivularia* sp. | NR_102526 | 98 | *Bacteria; Cyanobacteria; Nostocales; Rivulariaceae; Rivularia* |
| OTU221 | *Synechococcus* sp. | CP016483 | 91 | *Bacteria; Cyanobacteria; Synechococcales; Synechococcaceae; Synechococcus* |
| OTU391 | *Synechococcus* sp. | CP016483 | 96 | *Bacteria; Cyanobacteria; Synechococcales; Synechococcaceae; Synechococcus* |
| OTU258 | *Leptolyngbya* sp. | KC848639 | 91 | *Bacteria; Cyanobacteria; Synechococcales; Leptolyngbyaceae; Oculatella* |

**Table S2** Matrix of geographical distance (Km) among sites in this study

|  | EHLSa | EHLSb | EHLSc | QHLSa | QHLSb | QHLSc | TSLSa | TSLSb | TSLSc | GHLSa | GHLSb | GHLSc | XCDLSa | XCDLSb | XCDLSc | CKLSa | CKLSb | CKLSc |
| --- | --- | --- | --- | --- | --- | --- | --- | --- | --- | --- | --- | --- | --- | --- | --- | --- | --- | --- |
| EHLSa | 0.0 | 0.0 | 0.0 | 8.7 | 8.7 | 8.7 | 349.0 | 349.0 | 349.0 | 287.8 | 287.8 | 287.8 | 514.4 | 514.4 | 514.4 | 148.4 | 148.4 | 148.4 |
| EHLSb | 0.0 | 0.0 | 0.0 | 8.7 | 8.7 | 8.7 | 349.0 | 349.0 | 349.0 | 287.8 | 287.8 | 287.8 | 514.4 | 514.4 | 514.4 | 148.4 | 148.4 | 148.4 |
| EHLSc | 0.0 | 0.0 | 0.0 | 8.7 | 8.7 | 8.7 | 349.0 | 349.0 | 349.0 | 287.8 | 287.8 | 287.8 | 514.4 | 514.4 | 514.4 | 148.4 | 148.4 | 148.4 |
| QHLSa | 8.7 | 8.7 | 8.7 | 0.0 | 0.0 | 0.0 | 340.6 | 340.6 | 340.6 | 279.4 | 279.4 | 279.4 | 506.0 | 506.0 | 506.0 | 139.9 | 139.9 | 139.9 |
| QHLSb | 8.7 | 8.7 | 8.7 | 0.0 | 0.0 | 0.0 | 340.6 | 340.6 | 340.6 | 279.4 | 279.4 | 279.4 | 506.0 | 506.0 | 506.0 | 139.9 | 139.9 | 139.9 |
| QHLSc | 8.7 | 8.7 | 8.7 | 0.0 | 0.0 | 0.0 | 340.6 | 340.6 | 340.6 | 279.4 | 279.4 | 279.4 | 506.0 | 506.0 | 506.0 | 139.9 | 139.9 | 139.9 |
| TSLSa | 349.0 | 349.0 | 349.0 | 340.6 | 340.6 | 340.6 | 0.0 | 0.0 | 0.0 | 61.5 | 61.5 | 61.5 | 165.4 | 165.4 | 165.4 | 201.2 | 201.2 | 201.2 |
| TSLSb | 349.0 | 349.0 | 349.0 | 340.6 | 340.6 | 340.6 | 0.0 | 0.0 | 0.0 | 61.5 | 61.5 | 61.5 | 165.4 | 165.4 | 165.4 | 201.2 | 201.2 | 201.2 |
| TSLSc | 349.0 | 349.0 | 349.0 | 340.6 | 340.6 | 340.6 | 0.0 | 0.0 | 0.0 | 61.5 | 61.5 | 61.5 | 165.4 | 165.4 | 165.4 | 201.2 | 201.2 | 201.2 |
| GHLSa | 287.8 | 287.8 | 287.8 | 279.4 | 279.4 | 279.4 | 61.5 | 61.5 | 61.5 | 0.0 | 0.0 | 0.0 | 226.7 | 226.7 | 226.7 | 140.6 | 140.6 | 140.6 |
| GHLSb | 287.8 | 287.8 | 287.8 | 279.4 | 279.4 | 279.4 | 61.5 | 61.5 | 61.5 | 0.0 | 0.0 | 0.0 | 226.7 | 226.7 | 226.7 | 140.6 | 140.6 | 140.6 |
| GHLSc | 287.8 | 287.8 | 287.8 | 279.4 | 279.4 | 279.4 | 61.5 | 61.5 | 61.5 | 0.0 | 0.0 | 0.0 | 226.7 | 226.7 | 226.7 | 140.6 | 140.6 | 140.6 |
| XCDLSa | 514.4 | 514.4 | 514.4 | 506.0 | 506.0 | 506.0 | 165.4 | 165.4 | 165.4 | 226.7 | 226.7 | 226.7 | 0.0 | 0.0 | 0.0 | 366.5 | 366.5 | 366.5 |
| XCDLSb | 514.4 | 514.4 | 514.4 | 506.0 | 506.0 | 506.0 | 165.4 | 165.4 | 165.4 | 226.7 | 226.7 | 226.7 | 0.0 | 0.0 | 0.0 | 366.5 | 366.5 | 366.5 |
| XCDLSc | 514.4 | 514.4 | 514.4 | 506.0 | 506.0 | 506.0 | 165.4 | 165.4 | 165.4 | 226.7 | 226.7 | 226.7 | 0.0 | 0.0 | 0.0 | 366.5 | 366.5 | 366.5 |
| CKLSa | 148.4 | 148.4 | 148.4 | 139.9 | 139.9 | 139.9 | 201.2 | 201.2 | 201.2 | 140.6 | 140.6 | 140.6 | 366.5 | 366.5 | 366.5 | 0.0 | 0.0 | 0.0 |
| CKLSb | 148.4 | 148.4 | 148.4 | 139.9 | 139.9 | 139.9 | 201.2 | 201.2 | 201.2 | 140.6 | 140.6 | 140.6 | 366.5 | 366.5 | 366.5 | 0.0 | 0.0 | 0.0 |
| CKLSc | 148.4 | 148.4 | 148.4 | 139.9 | 139.9 | 139.9 | 201.2 | 201.2 | 201.2 | 140.6 | 140.6 | 140.6 | 366.5 | 366.5 | 366.5 | 0.0 | 0.0 | 0.0 |

**Table S3** GPS locations and the measured environmental parameters of the studied samples.

| Sample | GPS Location | Salinity (g/L) | pH | Water temperature (^o^C) | Turbidity (NTU) | Illumination intensity (KLUX) | DOC (mM) | Chlorophyll-a (ug/L) | DIC (mM) | TP (uM) | TN (uM) | Na | K | Mg | Ca | Cl | SO_4_^2-^ |
| --- | --- | --- | --- | --- | --- | --- | --- | --- | --- | --- | --- | --- | --- | --- | --- | --- | --- |
| EHLSa | 36^o^33.4'N/ 100^o^43.3'E | 0.8 | 8.7 | 11.1 | 11.7 | 73.5 | 2.6 | 21.4 | 7.8 | 8.9 | 97.7 | 253.2 | 13.0 | 89.9 | 14.4 | 299.6 | 146.7 |
| EHLSb | 36^o^33.4'N/ 100^o^43.3'E | 0.8 | 8.7 | 11.7 | 10.5 | 73.5 | 2.3 | 17.9 | 9.6 | 9.1 | 130.3 | 250.5 | 12.6 | 94.8 | 10.3 | 295.1 | 144.0 |
| EHLSc | 36^o^33.4'N/ 100^o^43.3'E | 0.8 | 8.6 | 11.6 | 10.2 | 73.5 | 1.9 | 17.8 | 8.2 | 8.8 | 108.9 | 250.0 | 12.8 | 92.5 | 15.4 | 296.6 | 147.4 |
| QHLSa | 36^o^33.3'N/ 100^o^37.5'E | 16.1 | 9.2 | 9.7 | 0.6 | 104.6 | 8.1 | 0.1 | 29.8 | 4.4 | 155.8 | 4298.8 | 270.8 | 1090.6 | 0.0 | 7482.1 | 2992.4 |
| QHLSb | 36^o^33.3'N/ 100^o^37.5'E | 16.6 | 9.0 | 9.6 | 0.6 | 104.6 | 9.1 | 0.2 | 29.8 | 4.6 | 167.8 | 4390.6 | 243.9 | 1345.1 | 0.0 | 7571.1 | 3000.2 |
| QHLSc | 36^o^33.3'N/ 100^o^37.5'E | 15.1 | 9.0 | 9.7 | 0.7 | 104.6 | 16.0 | 0.2 | 29.0 | 4.1 | 154.6 | 4074.5 | 239.6 | 776.4 | 0.0 | 7335.3 | 2674.4 |
| TSLSa | 37^o^11.6'N/ 96^o^53.3'E | 30.3 | 8.8 | 11.3 | 1.6 | 85.2 | 4.7 | 1.8 | 18.8 | 5.8 | 372.6 | 6812.8 | 130.5 | 1978.1 | 0.0 | 13532.1 | 7858.2 |
| TSLSb | 37^o^11.6'N/ 96^o^53.3'E | 29.0 | 8.9 | 11.5 | 1.7 | 85.2 | 4.4 | 1.6 | 24.2 | 4.3 | 441.1 | 6706.4 | 162.6 | 1978.6 | 0.0 | 12442.9 | 7702.3 |
| TSLSc | 37^o^11.6'N/ 96^o^53.3'E | 27.8 | 8.9 | 10.9 | 3.3 | 85.2 | 3.4 | 0.7 | 12.4 | 5.6 | 413.4 | 6181.8 | 325.5 | 1905.4 | 0.0 | 11908.1 | 7503.1 |
| GHLSa | 37^o^08.2'N/ 97^o^34.6'E | 69.1 | 7.8 | 13.8 | 5.0 | 101.5 | 3.0 | 1.6 | 8.4 | 5.6 | 264.4 | 18950.2 | 338.9 | 3487.3 | 0.0 | 35590.7 | 10758.5 |
| GHLSb | 37^o^08.2'N/ 97^o^34.6'E | 72.8 | 8.0 | 13.5 | 2.1 | 101.5 | 2.2 | 0.6 | 6.2 | 4.1 | 331.4 | 19448.0 | 436.5 | 2969.6 | 56.1 | 37634.9 | 12256.6 |
| GHLSc | 37^o^08.2'N/ 97^o^34.6'E | 73.3 | 7.8 | 13.4 | 3.8 | 101.5 | 2.7 | 0.8 | 7.0 | 5.9 | 329.1 | 22804.2 | 469.3 | 3264.9 | 0.0 | 37038.6 | 9760.9 |
| XCDLSa | 37^o^27.2'N/ 95^o^30.6'E | 138.3 | 8.2 | 11.5 | 14.4 | 95.0 | 3.7 | 5.3 | 13.6 | 4.4 | 254.9 | 43002.0 | 743.4 | 2904.7 | 0.0 | 63066.6 | 28566.6 |
| XCDLSb | 37^o^27.2'N/ 95^o^30.6'E | 139.3 | 8.3 | 11.3 | 17.4 | 95.0 | 3.7 | 2.5 | 14.0 | 4.4 | 219.0 | 43228.2 | 752.3 | 2911.3 | 0.0 | 63414.4 | 28958.0 |
| XCDLSc | 37^o^27.2'N/ 95^o^30.6'E | 140.6 | 8.3 | 11.1 | 15.4 | 95.0 | 3.8 | 2.4 | 14.2 | 4.8 | 216.8 | 43369.7 | 742.1 | 3032.8 | 0.0 | 64079.2 | 29399.6 |
| CKLSa | 36^o^45.1'N/ 99^o^04.8'E | 365.6 | 7.4 | 16.6 | 13.9 | 98.9 | 25.3 | 9.5 | 17.0 | 3.8 | 325.3 | 77447.5 | 4975.0 | 34842.0 | 224.2 | 213241.9 | 34888.9 |
| CKLSb | 36^o^45.1'N/ 99^o^04.8'E | 319.2 | 7.6 | 15.3 | 14.8 | 98.9 | 24.9 | 4.9 | 16.2 | 13.7 | 283.0 | 66611.0 | 4371.4 | 31554.4 | 231.9 | 185582.2 | 30857.0 |
| CKLSc | 36^o^45.1'N/ 99^o^04.8'E | 340.8 | 7.8 | 13.5 | 9.2 | 98.9 | 26.3 | 0.4 | 16.6 | 9.8 | 413.4 | 64754.9 | 5106.1 | 36991.7 | 413.8 | 195028.7 | 38541.1 |

**Fig. S1**


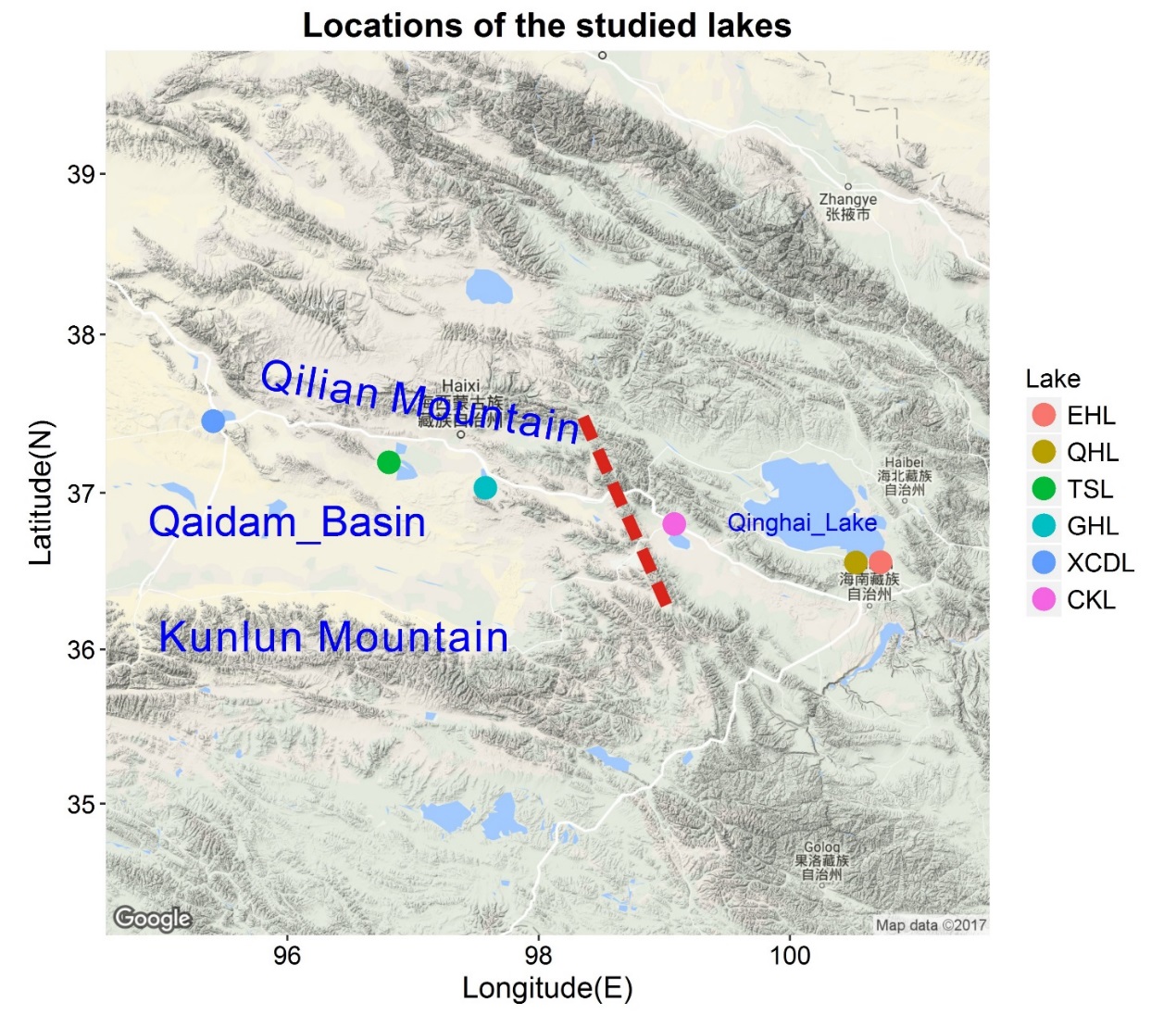


Fig. S2
